# Experiencing an Elongated Limb in Virtual Reality Modifies the Tactile Distance Perception of the Corresponding Real Limb

**DOI:** 10.1523/ENEURO.0244-23.2024

**Published:** 2024-06-14

**Authors:** François Le Jeune, Marco D’Alonzo, Valeria Piombino, Alessia Noccaro, Domenico Formica, Giovanni Di Pino

**Affiliations:** ^1^NEXT: Neurophysiology and Neuroengineering of Human-Technology Interaction Research Unit, Università Campus Bio-Medico di Roma, Rome 00128, Italy; ^2^Neurorobotics Lab, Newcastle University, Newcastle upon Tyne NE1 7RU, United Kingdom

**Keywords:** body representation, body schema, embodiment, reference frame, tactile distance perception, virtual reality

## Abstract

In measurement, a reference frame is needed to compare the measured object to something already known. This raises the neuroscientific question of which reference frame is used by humans when exploring the environment. Previous studies suggested that, in touch, the body employed as measuring tool also serves as reference frame. Indeed, an artificial modification of the perceived dimensions of the body changes the tactile perception of external object dimensions. However, it is unknown if such a change in tactile perception would occur when the body schema is modified through the illusion of owning a limb altered in size. Therefore, employing a virtual hand illusion paradigm with an elongated forearm of different lengths, we systematically tested the subjective perception of distance between two points [tactile distance perception (TDP) task] on the corresponding real forearm following the illusion. Thus, the TDP task is used as a proxy to gauge changes in the body schema. Embodiment of the virtual arm was found significantly greater after the synchronous visuotactile stimulation condition compared with the asynchronous one, and the forearm elongation significantly increased the TDP. However, we did not find any link between the visuotactile-induced ownership over the elongated arm and TDP variation, suggesting that vision plays the main role in the modification of the body schema. Additionally, significant effect of elongation found on TDP but not on proprioception suggests that these are affected differently by body schema modifications. These findings confirm the body schema malleability and its role as a reference frame in touch.

## Significance Statement

Evidence shows that humans use their body dimensions as a reference frame to perceive the dimensions of the objects in the environment when using touch. We employed a modified version of the virtual hand illusion (VHI) to induce embodiment of a fake elongated forearm. After the induction of the illusion, we tested if the perception of the distance between two points touching the real forearm changed. We show that the forearm elongation increases the perception of such distance. However, such elongation was not related to the embodiment level elicited by the VHI paradigm. These findings demonstrate the importance of the visual information in body schema modification, shedding new light on the relation between embodiment, body schema, and world perception.

## Introduction

Sensory feedback is exploited to experience the environment and guides physical interaction. In a nutshell, a parameter to be measured requires a unit of measure and a reference frame to which compare the measure. Especially, when the environment is experienced through our body (i.e., in somatosensation), it has been suggested that the reference frame we employ is the metric properties of our body (Proffitt and Likenauger, 2013; Harris et al., 2015). This hypothesis is sound because it has been widely shown that the brain integrates a higher-order reconstruction of the body that is an implicit model of its metric properties (Longo and Haggard, 2010, 2012), namely, the body schema (De Vignemont, 2010), and of the space where it interacts. This also means that change in the body schema could affect the perception of the environment.

In touch, the perception of the distance between two stimulated points, i.e., tactile distance perception (TDP), depends on the part of the body which is stimulated. Such phenomenon, known as Weber’s illusion (Weber, 1996), is certainly linked to the different tactile receptor densities of different body parts, but there is more: for instance, a tactile receptor density variation of 340% results in a variation of only 30% in TDP between the palm of the hand and the forearm (Weinstein, 1968; Green, 1982). Hence, besides receptor density, a subsequent neural process must be involved in Weber’s illusion. This process is a rescaling operation to compensate for the different receptor densities, and it is likely to be based on a representation of the body parts in the body schema. Furthermore, since the body schema is continuously updated by the multimodal sensory input (De Vignemont, 2010; Longo et al., 2010; Serino and Haggard, 2010; Romano et al., 2021), we hypothesize that a modification of the body schema achieved through multisensory feedback modulation would induce a consequent change of TDP. It has indeed been shown that by visually deforming the hand and forearm for ∼1 h, the TDP relative to the participant's forearm would change with respect to the not altered control part (Taylor-Clarke et al., 2004). In another experiment inspired by the Pinocchio illusion (Lackner, 1988), the proprioceptive illusion of an elongation of the index finger significantly increased the TDP on the same finger. The illusion of finger elongation was achieved by stimulating the spindles of the right arm biceps with a vibrator placed on the tendon (known as tendon-vibration illusion, Pinardi et al., 2020), while participants held their left index finger with their right arm (De Vignemont et al., 2005). Those studies confirmed that the perceived size of the body impacts on TDP, in line with the hypothesis that the body schema acts as a reference frame for touch.

Another strategy to modify the perceived size of the body exploits the embodiment of the fake hand through the rubber hand illusion (RHI) paradigm (Botvinick and Cohen, 1998), while the fake hand is placed in an artificial position, farther than the real hand, inducing the illusory feeling of having a longer arm (Armel and Ramachandran, 2003; Kilteni et al., 2012; Kalckert et al., 2019). Interestingly, previous works found correlations between the changes in perception of object dimensions and the perceived embodiment, induced by a RHI paradigm, of a full body or a rubber hand with rescaled dimensions, proving the existence of a link between a bodily illusion induced by the embodiment of external body part and the perception change (Van Der Hoort et al., 2011; Bruno and Bertamini, 2010).

However, to our knowledge, no previous study has investigated the possible change in TDP following a modification of the body schema through the embodiment of a fake limb. Thus, we used a RHI paradigm in virtual reality (VR), a virtual hand illusion (VHI), with an elongated virtual forearm to induce in participants the feeling of owning a distorted limb and investigated the evolution of the TDP on the corresponding real body part.

Following the findings of Taylor-Clarke et al. (2004) and De Vignemont et al. (2005) who, using bodily illusion, found that increased body size perception increases TDP, and in contrast with Canzoneri et al. (2013) showing that tool-use reduces TDP, we hypothesized that the synchronous brush stroking of a hand at the end of an elongated forearm would have increased the TDP on the corresponding real forearm and that the variation in TDP would have positively correlated with the forearm elongation and with the achieved level of embodiment of the hand. The asynchronous stimulation has been acquired as control condition.

## Materials and Methods

### Participants

Sixty-nine participants (33 females, six left-handed, aged 23.9 ± 5.6 years) were enrolled in the study. For one of our analyses, participants were divided into three independent groups, depending on the first condition they would undergo (three conditions tested); thus each group contains 23 participants (see below, Data analysis, for detailed explanation). This number of enrolled participants per group has been based on the TDP task (TDPT) data distribution from the study of Taylor-Clarke et al. (2004) to show a 7% mean shift in TDP between pre- and post-VHI, achieving an effect size of 0.32, a power superior to 0.8, and considering an independent *t* test. All participants reported to have normal tactile sensation of the hand, forearm, and forehead and normal or corrected-to-normal vision. All participants provided written informed consent before the experiment in accordance with the Declaration of Helsinki and following amendments. The experiment was conducted after the approval of the Ethics Committee of Università Campus Bio-Medico di Roma.

### Setup

**Figure 1.**
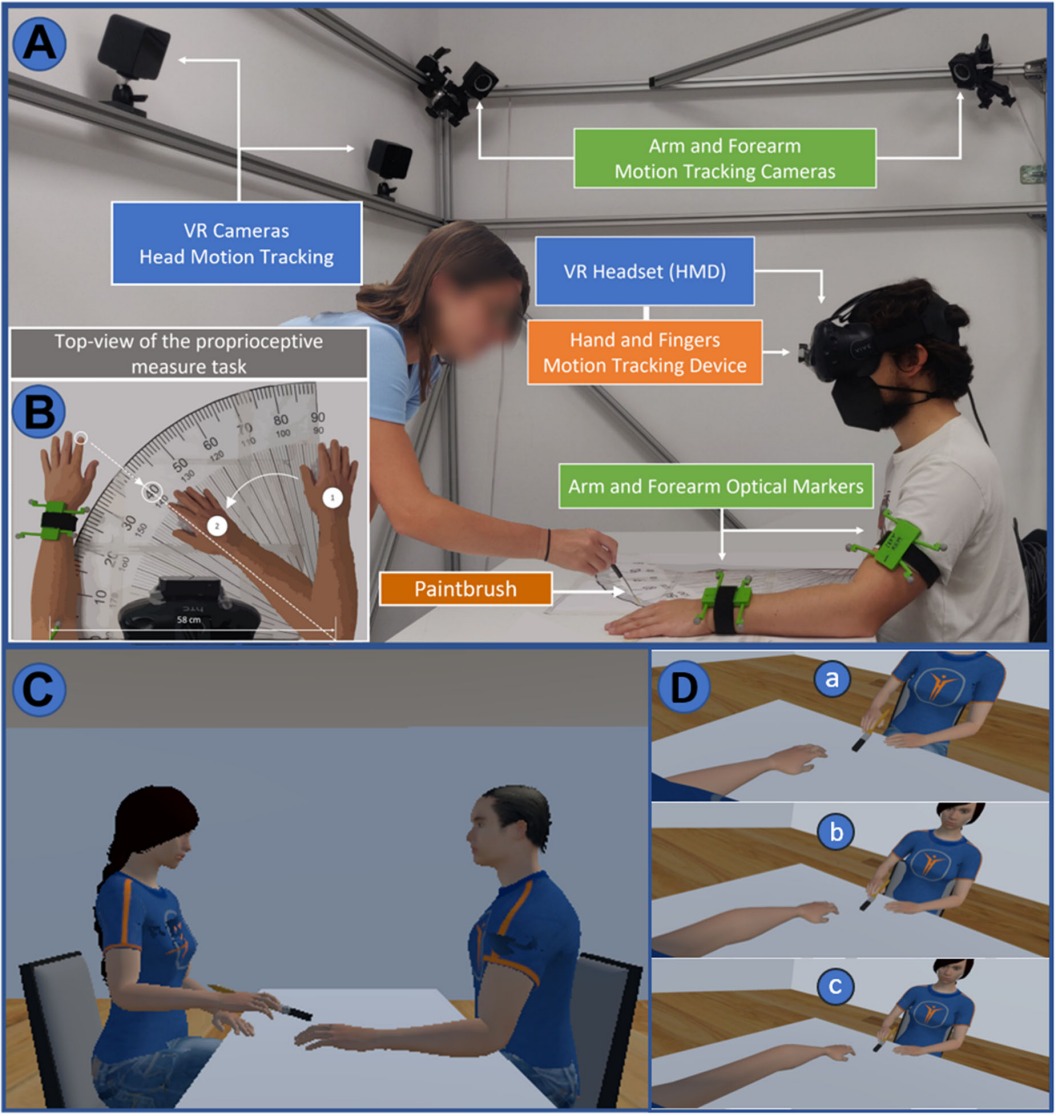
Real-world and virtual experimental setup. ***A***, An experimental setup with the experimenter (left) and participant (right) in default position. ***B***, A PD measurement technique. ***C***, Virtual environment,—sideview. The virtual experimenter (left) holding the paintbrush and the participant avatar (right) in default position, without forearm elongation. ***D***, Virtual environment—participant's 1PP. Without forearm elongation (***a***), with 20 cm elongation (***b***), with 40 cm elongation (***c***).

The participant sat comfortably on a chair in front of a table placed within a 2.40 m × 2.00 m × 1.80 m metallic structure. A large paper goniometer (58 cm radius) was displayed upon the table. Participants visualized the immersive virtual environment through a VR system (HTC Vive, HTC). They wore a VR headset (head-mounted device, HMD), and the HMD movement was tracked by two infrared cameras (base stations). To enhance the immersion of participants inside the virtual environment and their sense of agency over the virtual upper limb, the movements of their real left arm, forearm, hand, and fingers were tracked by motion capture systems. Arm and forearm movements were tracked with four infrared cameras (OptiTrack 13W, NaturalPoint) and reflective optical markers worn by the participant, whereas finger and hand movements were tracked by a dedicated infrared motion tracking device attached on the HMD (Leap Motion, Ultraleap). We developed a VR environment (using the game engine Unity, version 2018.3.0, Unity Technologies) which replicates the lab room where the experiment was run, including the table and the chair. In the virtual environment, participants saw in a first-person perspective (1PP) their avatar's body (male or female) sitting in front of the virtual table. In the default position, both the participant and their avatar had the left forearm (palm down) on the table and the right arm alongside their body. The left forearm of the participant avatar could be elongated by different lengths (20 or 40 cm depending on the condition). The real experimenter, located in front of the participant, stimulated the participant's left-hand index finger with a paintbrush. Both the experimenter and the paintbrush movement were replicated in the virtual environment (Fig. 1). The virtual avatars were created with the open-source software MakeHuman.

### Experiment

#### General procedure

We investigated the effects of a VHI with an elongated virtual forearm on TDP under three conditions. Synchronous VHI were performed with the virtual forearm elongated by 20 cm (20S) or 40 cm (40S), whereas an asynchronous VHI with a 20 cm forearm elongation (20A) was used as control condition. We included only a single common control condition based on asynchronous tactile stimulation of the virtual hand. This experimental choice did not allow to apply a balanced factorial design to our study, but this choice has been made to reduce the duration of the experimental sessions for participant comfort and, consequently, reliability of the collected data and preventing the habituation to VHI (Bekrater-Bodmann et al., 2012; Convento et al., 2018).

Firstly, the virtual environment was turned pitch black, and participants underwent a preliminary task of TDP (pre-TDPT) to measure their baseline TDP (see below, Measures, for detailed information). Then, when VR was activated, participants were instructed to look at their surroundings (moving only their head) to familiarize with the virtual environment. To give participants agency over the avatar, they were asked to move their left arm for 90 s and then their hand and fingers for additional 90 s while looking at their virtual counterparts which moved accordingly.

**Figure 2.**
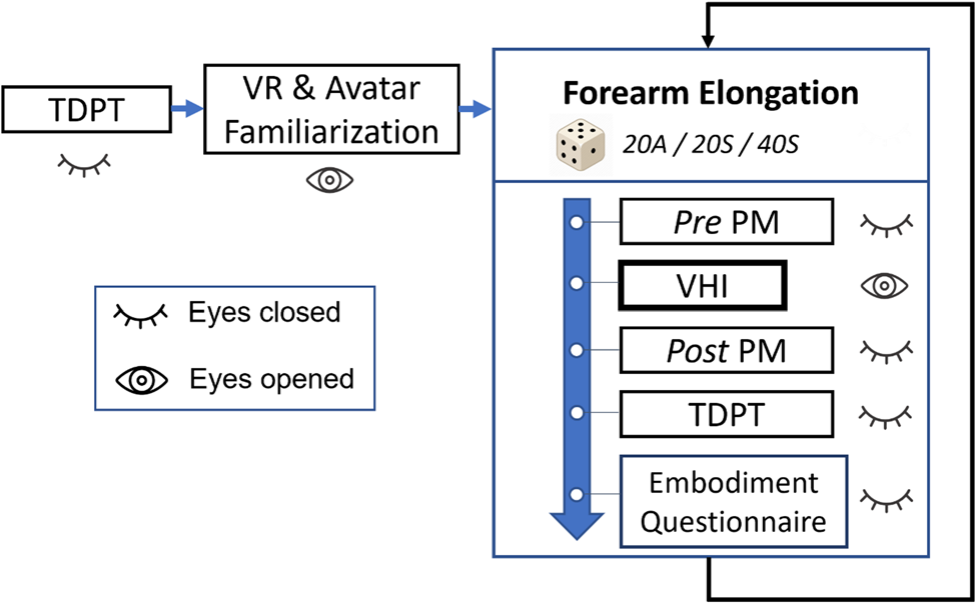
Experimental protocol of the experiment.

Finally, participants were instructed to place back their left arm in the default position and to keep it still until further notice. The VR environment was turned pitch black. Participants then performed a proprioceptive measure (pre-PM), in which they had to indicate the felt position of the tip of their left index finger (see below, Measures, for detailed information). The left virtual forearm was then elongated by 20 or 40 cm depending on the condition. The virtual environment was illuminated again, and participants were asked to look at and pay close attention to the (virtual) hand. To perform the VHI, the experimenter started the brush stroking synchronously or asynchronously (depending on the condition) with respect to the visual virtual brush stroking performed by the virtual experimenter on the left index finger of the participant avatar. During the asynchronous condition, the tactile stimulation on the real hand was delayed from the visual stimulation by 1 s to ensure that both stimuli are not integrated (Maselli et al., 2016). Brush stroking lasted 90 s. Following the VHI, post-PM was immediately performed, followed by a post-TDPT. Participants then were asked to answer a questionnaire evaluating the strength of the embodiment illusion elicited by the VHI. The latest steps, starting from the PM prior to the VHI, were repeated for every VHI condition, and conditions were tested in a pseudorandom order among participants (Fig. 2). The whole procedure, including the preparation of the participant, lasted ∼1.5 h.

#### Measures

To measure the embodiment outcomes following the VHI, we asked participants to fill a self-evaluation questionnaire adapted from Botvinick and Cohen (1998; D’Alonzo et al., 2019) to evaluate the strength of the ownership illusion over the virtual hand (Table 1). Three of the statements (Table 1, Q1, Q2, and Q3) were ownership-related and referred to the extent of sensory transfer into the virtual hand and its self-attribution during the VHI. The six other statements (Table 1, Q4, Q5, Q6, Q7, Q8, and Q9) served as control items to assess compliance, suggestibility, and placebo effect. For each statement, participants were asked to rate the extent to which these statements did or did not apply to their experience, by using a seven-point Likert scale. On this scale, −3 meant “I am absolutely certain it did not apply,” 0 meant “uncertain whether it applied or not,” and +3 meant “I am absolutely certain it applied.” The statements were presented to participants in a random order. The embodiment outcome of the VHI was taken from the questionnaire results and computed as the RHI index. The RHI index is defined as the difference between the mean score of the ownership statements and the mean score of the control statements (Abdulkarim and Ehrsson, 2016). The greater the RHI index, the stronger the perceived embodiment.

**Table 1.** Embodiment questionnaire with embodiment and control statements

VHI embodiment questionnaire		
Embodiment statements	Q1	It seemed like I felt the touch of the paintbrush on the spot on which I was seeing the hand being touched
	Q2	It seemed like the touch I was feeling were due to the touch of the paintbrush on the hand I was seeing
	Q3	It felt like the hand I was seeing were my own hand
Control statements	Q4	It felt like my real hands were moving toward the hand I was seeing
	Q5	It felt like I had three arms or three hands
	Q6	It felt like the touch I was perceiving were coming from somewhere between my hand and the hand I was seeing
	Q7	It felt like my real hands were turning virtual
	Q8	It felt like the hand I was seeing was moving toward my real hand
	Q9	The hand I was seeing was starting to look like my real hand, in terms of shape, skin tone, freckles, or other characteristics

To measure the proprioceptive drift (PD) caused by the VHI (Botvinick and Cohen, 1998), participants were helped placing their right arm on the paper goniometer, parallelly to the left arm, in the starting position (90°). Participant forearms were always positioned on the extremities of the goniometer so that their elbows in-between distance would be of 580 mm (Fig. 1B). They were instructed to point with their right index finger toward the felt position of the tip of their left index finger by flexing the right forearm while keeping the forearm, hand, and finger along a straight line (Fig. 1B). We collected the corresponding angle and helped participants placing their right arm back alongside their body. Angular values were converted into the left elbow-index perceived distance expressed in millimeters, using a simple trigonometric function to obtain the elbow-index distance corresponding to the given angular value (*θ*; Eq. 1.1). The PM was performed right before (pre-PM) and after (post-PM) every VHI, and the resulting PD was calculated as the difference between the post-PM and the pre-PM (Eq. 1.2) as follows:

(1.1) $$\begin{array}{c} \mathrm{PM}=580\times \tan\;(\theta ), \end{array}\;$$

(1.2) $$\begin{array}{c} \mathrm{PD}=PM_{\mathrm{Post}}-\;PM_{\mathrm{Pre}}. \end{array}$$

**Figure 3.**
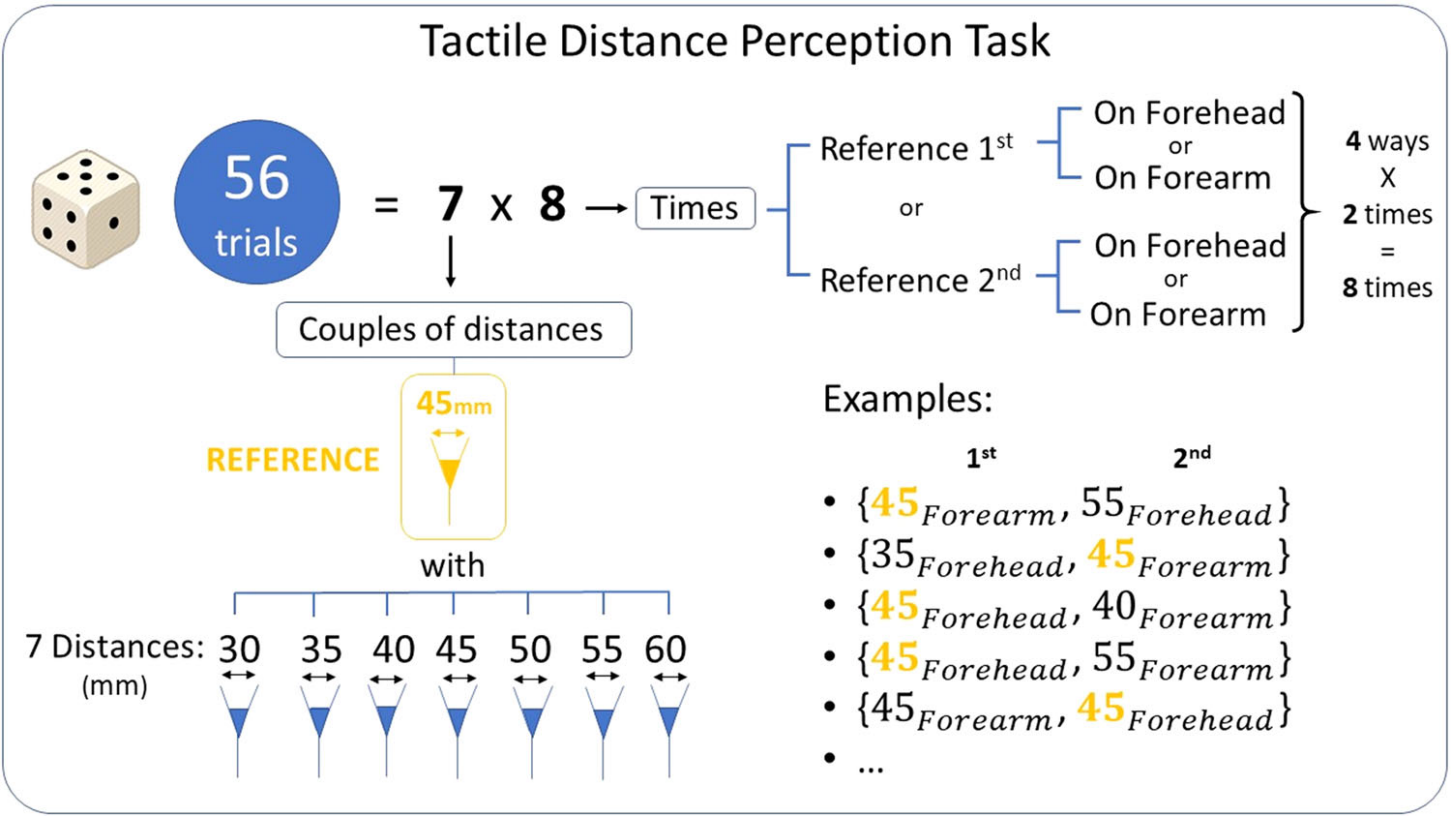
TDPT. Seven couples of distances tested in four different ways (28 different trials); each way repeated twice: 56 trials in total.

To measure the TDP, participants underwent a TDPT. During the TDPT, blindfolded participants received 56 couples of tactile stimulation: one on the forearm (investigated TDP) and one on the forehead (used as reference TDP) in a random order. Tactile stimulations were performed using fork-like tools composed of two blunt tips with a specific distance between each other (Fig. 3). After each couple of stimulation, participants were asked to report in which of the two stimulations the distance between the tips of the forks was felt larger: they were instructed to answer “one” if the first stimulation was felt larger, “two” if it was the second. We recorded the corresponding body part on which the stimulation distance was felt larger. Distances between the tips of the forks were chosen based on a previous study using a similar task (Taylor-Clarke et al., 2004). In all couples of stimulations, we used a reference fork 45 mm wide and a fork with 30, 35, 40, 45, 50, 55, or 60 mm between-tips distance. The order, body part, and distance of the stimulations were randomized and balanced among the 56 couples of stimulations (28 unique couples of stimulations, each one performed twice; Fig. 3).

From the participants’ responses, we calculated the percentage of “forearm” answers (%ForeAns) for each difference (Δ*L*, in millimeters) between the length of the forearm stimulation (*L*_forearm_) and the length of the forehead stimulation (*L*_forehead_), positive values meaning bigger distance administered to the forearm (Eq. 2) as follows:

(2) $$\Delta \begin{array}{c} L=\;L_{\mathrm{forearm}}-\;L_{\mathrm{forehead}}, \end{array}$$

$$\begin{array}{c} \Delta L\;\in \{-15,\;-10,\;-5,\;0,\;5,\;10,\;15\} \end{array}.$$

**Figure 4.**
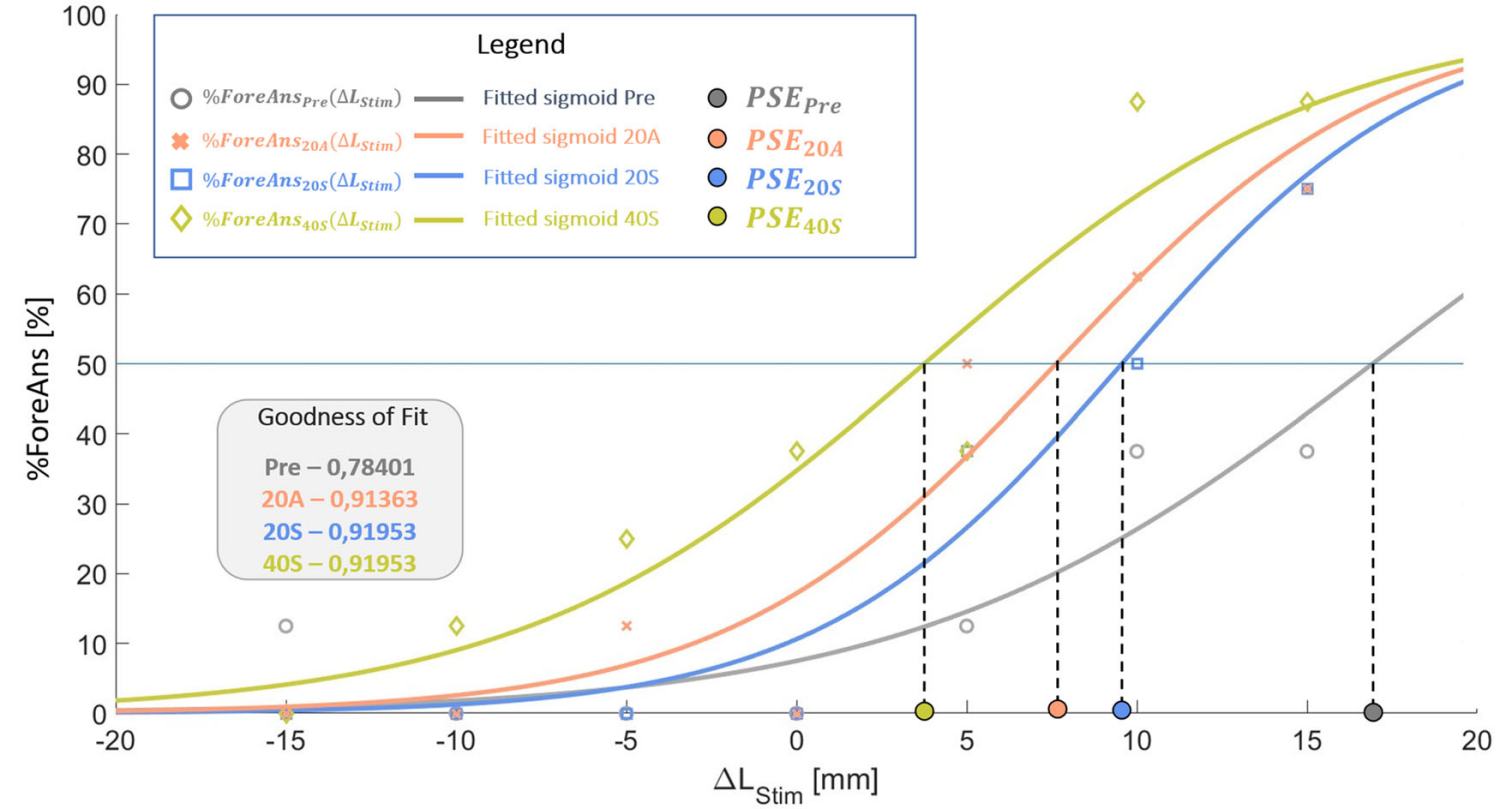
An example of a typical TDPT result plot for one subject. Raw results are the percentages of “forearm” answers in function of stimulation distance difference between the stimulation on the forearm and the stimulation on the forehead (Δ*L*). They are identified with gray empty circles (Pre), red crosses (20A), blue squares (20S), and green diamonds (40S). Continuous curves are their color-corresponding fitted sigmoid curves with their goodness-of-fit values specified in the top-left–hand corner. Filled circles on the abscissa indicate the graphical definition of the PSE.

The TDP was measured by the point of subjective equality (PSE, in millimeters). As clearly defined in Vidotto et al. (2019), “the point of subjective equality is any of the points along a stimulus dimension (here Δ*L*) at which a variable stimulus (here forearm stimulation distance) is judged by an observer to be equal to a standard stimulus (here forehead stimulation distance).” If the PSE is positive, it means that the stimulation distance on the forearm has to be greater (by “PSE” millimeters) than the stimulation distance on the forehead to be felt as equal to the stimulation distance on the forehead and vice versa for negative PSE. Thus, a reduction of PSE means the actual forearm stimulation distance has to be “less larger” than the forehead stimulation than before to be felt equal to the forehead stimulation, hence meaning that is was perceived larger. To calculate the PSE, we proceeded as follows: for every participant and every TDPT (Pre, 20A, 20S, 40S), we plotted (Fig. 4) the %ForeAns (*y*-axis) in function of the Δ*L* (*x*-axis) as independent variable and fitted the data distribution with the following psychophysics sigmoid function (Eq. 3.1):

(3.1) $$\begin{array}{c} P(\Delta L,\;\mathrm{PSE},\;\mathrm{EA})=\;\frac{100}{1+\exp\left(-\frac{\Delta L-\mathrm{PSE}}{0.5\times \mathrm{EA}}\right)}, \end{array}$$

adapted from Di Pino et al. (2020) where *P* is the probability (expressed as a percentage) of feeling the larger stimulation of the forearm. From the plotted curve (Fig. 4), the PSE can be defined as follows:

(3.2) $$\begin{array}{c} \mathrm{PSE}=\;\Delta L{|}_{P_{50\text{\%}}} \end{array}.$$

It represents the Δ*L* value of the point in which the curve corresponds to the *P* = 50% value, i.e., the Δ*L* value for which the participant has the same probability to perceive the larger stimulation on the forearm as on the forehead (perceived equality of distances). EA is the esteem accuracy and represents the Δ*L* value of the point in which the line tangent to the curve at the point of coordinates (PSE, 50%) reaches the value *P* = 100%, subtracted by the PSE value. It is the inverse of twice the slope of the curve at (PSE, 50%). Considering that we expected a change in TDP and not in tactile accuracy, the EA was not further considered in the study.

PSE values were obtained from the fittings results. The goodness-of-fit (*R*-squared) of all analyzed participants was above 0.6 (moderate effect size, Moore et al., 2013).

Then, we computed for each condition the variation of PSE, i.e., ΔPSE, the difference between the post-PSE (after the VHI) and the pre-PSE (baseline PSE for the first tested condition and PSE after the previous condition for following conditions).

### Data analysis

We firstly planned a general within-subject analysis (i.e., repeated measures). We considered the possibility that by undergoing different conditions successively without a pause between them, one condition could have an influence on the successive one and that by repeating the VHI, its intensity might decrease. Therefore, to investigate the order effect, i.e., the order in which the conditions were presented to participants on our outcomes and to counterbalance the lack of a 2-by-2 factorial design (no 40A condition), we ran a linear mixed model (LMM) considering the effects of synchronicity, elongation, and order. We expected an influence of the order effect, i.e., a predominant effect of the first condition presented to participants, and to discard its aforementioned effects from the analysis, a between-subject analysis was planned regarding the data relative to the first condition presented to participants only. Thus, three independent groups (of 23 subjects each) were formed depending on the first condition tested. In both analyses, the significance threshold was set to *p* values <0.05 for all statistical tests. Correlation analyses were performed between all three outcomes, and for each outcome, we pooled together data from all conditions. All statistical tests were conducted with JASP (JASP Team, 2023).

#### General analysis

For each condition, all data (RHI index, PD, and ΔPSE) were distributed normally (Shapiro–Wilk test, *p* > 0.05). We used a LMM on each of our outcomes. For each LMM, we introduced the outcome as the dependent variable; included fixed effects of synchronicity, elongation, and order; and included subjects as random effect. Post hoc tests were performed through the study of the contrasts (Bonferroni-corrected) between the conditions of interest. We tested the significance of the variations of PD and ΔPSE with respect to 0 using one-sample Student’s *t* tests. To investigate any difference of distribution in premeasures between conditions (20S vs 20A and 40S vs 20S) for the PD and ΔPSE, i.e., pre-PM and pre-PSE, we used Student's paired *t* tests on pre-PM data (normally distributed among conditions, Bonferroni-corrected for two comparisons) and Wilcoxon's signed-ranked *t* tests on pre-PSE data (non-normally distributed among conditions, Bonferroni-corrected for two comparisons). For correlation analyses, the data were pooled across conditions. Spearman's *ρ* was calculated between RHI index and PD and between RHI index and ΔPSE (RHI index data non-normally distributed), and Pearson's *r* was calculated between PD and ΔPSE.

#### First-condition analysis

Among each group, all data (RHI index, PD, ΔPSE) were normally distributed (Shapiro–Wilk test, *p* > 0.05). We used preplanned independent Student's *t* tests to evaluate the effects of synchronicity (20S vs 20A) and elongation (40S vs 20S) on RHI index, PD, and ΔPSE (Bonferroni-corrected for two comparisons). We tested the significance of the variations of PD and ΔPSE with respect to 0 using one-sample Student’s *t* tests. Independent Student's *t* tests were used to investigate any difference of distribution in pre-PM and pre-PSE data between conditions (normally distributed among conditions, Bonferroni-corrected for two comparisons). For correlation analyses, Pearson's *r* was calculated between all outcomes.

#### Data and code accessibility

Data and analysis code are publicly available and can be accessed at https://osf.io/fp75s/?view_only=b2be227e01f34f0383089303b974c1a0. The analyses were performed using MATLAB on a Windows 10 operation system.

## Results

A summary of the results can be found in Tables 2–7 with corresponding statistical values.

**Table 2.** Statistical table of the LMM used for the general analysis with corresponding degrees of freedom (df), *F* statistic values (*F*), and *p* values

Outcome	Effect	df	*F*	*p* value
RHI index	Synchronicity	1, 134.00	93.928	<0.001***
RHI index	Elongation	1, 134.00	0.004	0.947
RHI index	Order	2, 134.00	7.247	0.001**
PD	Synchronicity	1, 160	3.847	0.052
PD	Elongation	1, 160	0.837	0.362
PD	Order	2, 160	3.944	<0.021*
ΔPSE	Synchronicity	1, 190	1.517	0.220
ΔPSE	Elongation	1, 190	5.223	0.023*
ΔPSE	Order	2, 190	12.015	<0.001***

Extended data related to the LMM on the ΔEA outcome can be found in Extended Data Table 2-1.

* *p* < 0.05.

** *p* < 0.01.

*** *p* < 0.001.

10.1523/ENEURO.0244-23.2024.t2-1 Table 2-1 Download Table 2-1, DOCX file.

**Table 3.** Statistical table of the contrasts analyses from the LMM (Table 2) with corresponding estimates, standard errors (SE), confidence intervals (95% CI lower and upper), *z* statistics values (*z*) and *p* values

Outcome	Comparison	Estimate	SE	95% CI (lower)	95% CI (upper)	*z*	*p* value
RHI index	20S vs 20A	5.237	0.540	4.178	6.296	9.692	<0.001***
RHI index	40S vs 20A	5.201	0.541	4.141	6.261	9.619	<0.001***
RHI index	1st vs 2nd	2.439	0.720	1.027	3.851	3.386	0.004**
RHI index	1st vs 3rd	2.307	0.721	0.894	3.720	3.200	0.008**
RHI index	2nd vs 3rd	−0.133	0.721	−1.546	1.280	−0.184	1.000
PD	1st vs 2nd	207.965	75.085	60.800	355.130	2.770	0.017*
PD	1st vs 3rd	134.197	75.085	−12.968	281.361	1.787	0.222
PD	2nd vs 3rd	−73.768	75.085	−220.933	73.396	−0.982	0.978
ΔPSE	40S vs 20A	−2.186	2.069	−6.242	1.870	−1.056	1.000
ΔPSE	40S vs 20S	−4.729	2.069	−8.785	−0.673	−2.285	0.111
ΔPSE	1st vs 2nd	−11.207	2.753	−16.603	−5.810	−4.070	<0.001***
ΔPSE	1st vs 3rd	−12.136	2.759	−17.544	−6.728	−4.398	<0.001***
ΔPSE	2nd vs 3rd	−0.929	2.759	−6.337	4.478	−0.337	1.000

* *p* < 0.05.

** *p* < 0.01.

*** *p* < 0.001.

**Table 4.** Statistical table of the one-sample student *t* tests analyses from the general analysis with corresponding degrees of freedom (df), Cohen’s *d* (as effect size), SE (standard error) of Cohen's *d*, confidence intervals of Cohen's *d* (95% CI lower and upper), *t* statistics (*t*), and *p* values

Outcome	Condition	df	Cohen's *d*	SE Cohen's *d*	95% CI (lower)	95% CI (upper)	*t*	*p* value
PD	20A	54	0.004	0.135	−0.260	0.268	0.030	0.976
PD	20S	54	0.420	0.141	0.142	0.694	3.112	0.003**
PD	40S	54	0.456	0.142	0.176	0.732	3.382	0.001**
ΔPSE	20A	64	−0.213	0.125	−0.458	0.034	−1.717	0.091
ΔPSE	20S	64	0.039	0.124	−0.204	0.282	0.316	0.753
ΔPSE	40S	64	−0.285	0.127	−0.532	−0.036	−2.295	0.025*

* *p* < 0.05.

** *p* < 0.01.

*** *p* < 0.001.

**Table 5.** Statistical table of the independent student's *t* tests of the first-condition analysis with corresponding degrees of freedom (df), Cohen’s *d* (as effect size), SE (standard error) of Cohen's *d*, confidence intervals of Cohen's *d* (95% CI lower and upper), *t* statistics (*t*), and *p* values

Outcome	Effect (comparison)	df	Cohen's *d*	SE Cohen's *d*	95% CI (lower)	95% CI (upper)	*t*	*p* value
RHI index	Synchronicity (20S vs 20A)	44	1.024	0.331	0.403	1.635	3.473	0.002**
RHI index	Elongation (40S vs 20S)	44	0.179	0.296	−0.757	0.402	0.606	1.000
PD	Synchronicity (20S vs 20A)	42	0.232	0.304	−0.362	0.824	0.770	0.892
PD	Elongation (40S vs 20S)	43	0.298	0.302	−0.884	0.292	−0.999	0.646
ΔPSE	Synchronicity (20S vs 20A)	43	0.029	0.298	−0.556	0.613	0.096	1.000
ΔPSE	Elongation (40S vs 20S)	41	−0.778	0.327	−1.395	−0.153	−2.551	0.030*

Extended data related to the independent Student's *t* tests on the ΔEA outcome can be found in Extended Data Table 5-1.

* *p* < 0.05.

** *p* < 0.01.

*** *p* < 0.001.

10.1523/ENEURO.0244-23.2024.t5-1 Table5-1 Download Table5-1, DOCX file.

**Table 6.** Statistical table of the one-sample student *t* tests of the first-condition analysis with corresponding degrees of freedom (df), Cohen’s *d* (as effect size), SE (standard error) of Cohen's *d*, confidence intervals of Cohen's *d* (95% CI lower and upper), *t* statistics (*t*), and *p* values

Outcome	Condition	df	Cohen's *d*	SE Cohen's *d*	95% CI (lower)	95% CI (upper)	*t*	*p* value
PD	20A	22	0.571	0.225	0.123	1.007	2.737	0.012*
PD	20S	22	0.740	0.235	0.270	1.196	3.547	0.002**
PD	40S	22	0.650	0.229	0.193	1.095	3.116	0.005**
ΔPSE	20A	22	−0.544	0.223	−0.977	−0.100	−2.608	0.016*
ΔPSE	20S	21	−0.488	0.226	−0.926	−0.040	−2.288	0.033*
ΔPSE	40S	20	−1.360	0.303	−1.949	−0.753	−6.232	<0.001***

* *p* < 0.05.

** *p* < 0.01.

*** *p* < 0.001.

**Table 7.** Statistical table of the correlation analyses of both general and first-condition analyses with corresponding Pearson's *r* values or Spearman's *ρ* values, Fisher's *z* (as effect size), confidences intervals (95% CI lower and upper), and *p* values

Analysis	Outcomes	Data distrib.	Coef.	*r* or *ρ*	Fisher's *z*	95% CI (lower)	95% CI (upper)	*p* value
General	RHI index - PD	Non-normal (RHI index)	*ρ*	0.187	0.189	0.035	0.331	0.016*
General	RHI index - ΔPSE	Non-normal (RHI index)	*ρ*	−0.125	−0.126	−0.267	0.021	0.094
General	PD - ΔPSE	Normal	*r*	−0.056	−0.056	−0.213	0.103	0.488
1st condition	RHI index - PD	Normal	*r*	0.054	0.054	−0.189	0.290	0.666
1st condition	RHI index - ΔPSE	Normal	*r*	−0.225	−0.229	−0.443	0.018	0.069
1st condition	PD - ΔPSE	Normal	*r*	0.029	0.029	−0.219	0.273	0.821

* *p* < 0.05.

### General analysis

**Figure 5.**
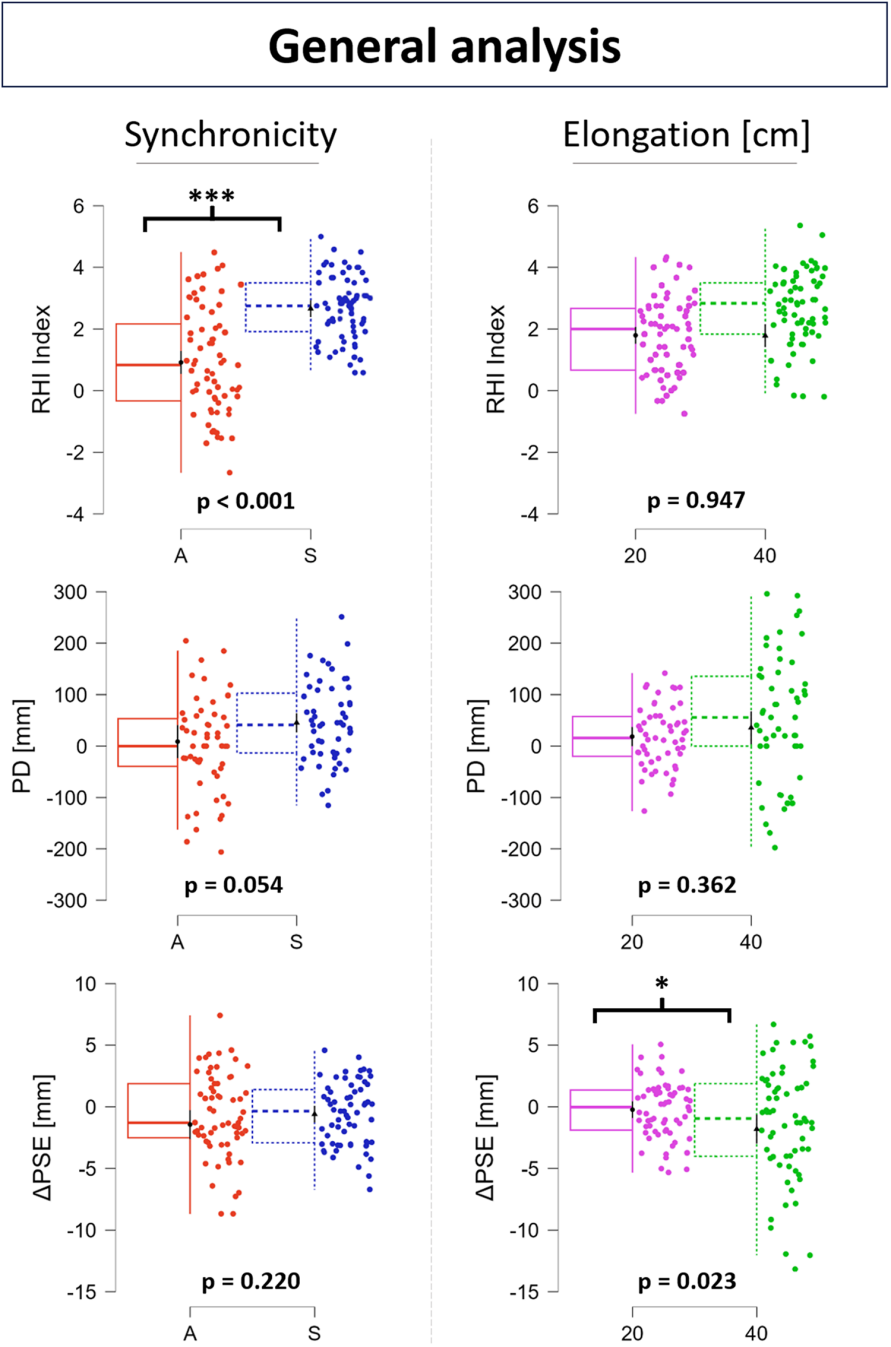
Box-jitter plots showing results of the general analysis (LMM) on the synchronicity effect (left) and elongation effect (right) on the RHI index (top), PD (middle), and ΔPSE (bottom). *P* values refer to the specific effect on the specific outcome. Asterisks meanings: *p < 0.05; **p < 0.01; ***p < 0.001. Extended data related to the ΔEA is displayed in Extended Data Figure 5-1.

**Figure 6.**
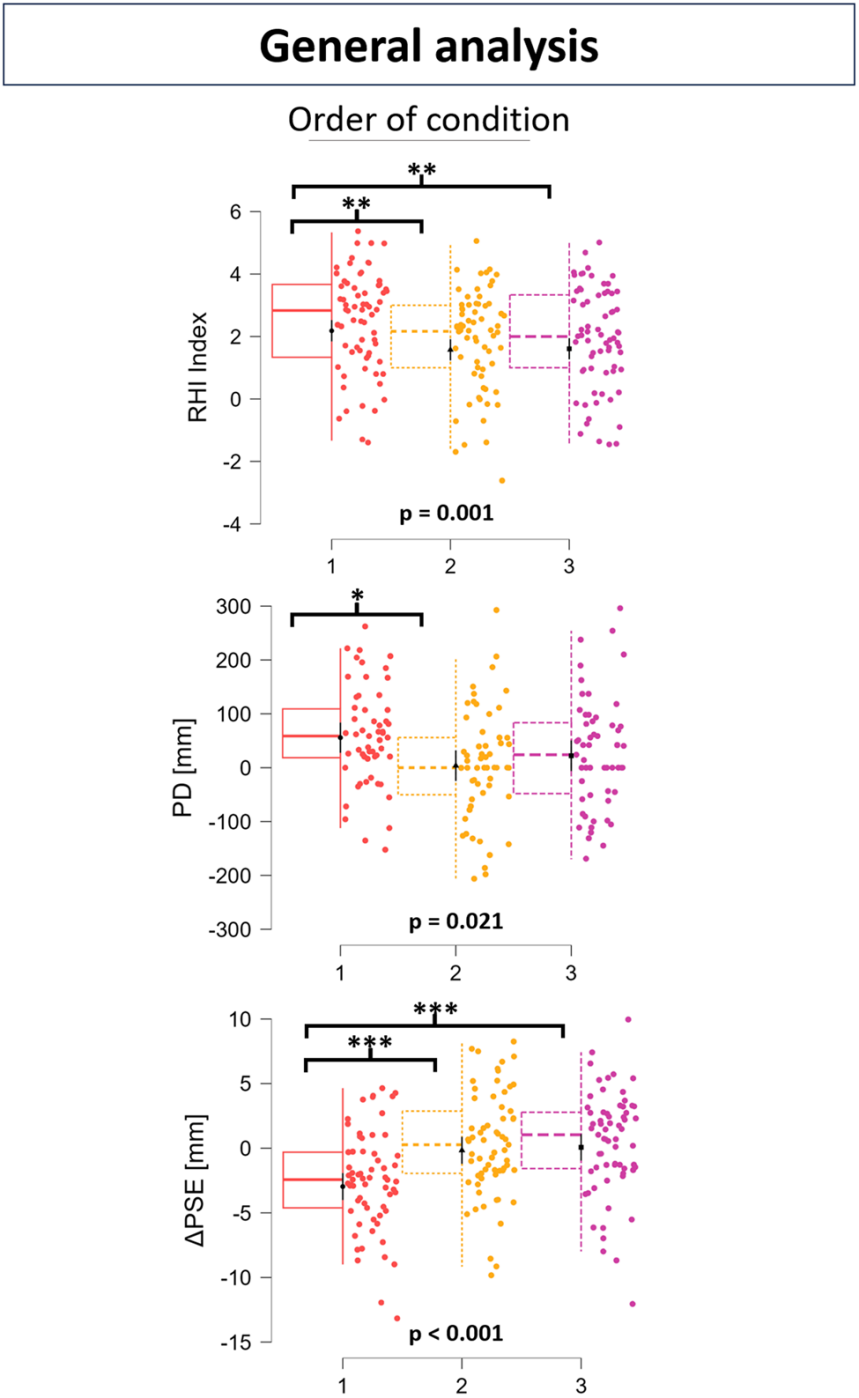
Box-jitter plots showing results of the general analysis (LMM) on the order of the condition presented to participants on the RHI index (top), PD (middle), and ΔPSE (bottom). *P* values refer to the order effect on the specific outcome. Asterisks meanings: *p < 0.05; **p < 0.01; ***p < 0.001.

Considering the RHI index, there was a significant effect of synchronicity (Fig. 5, top-left graph; df = 1, 134.00; *F* = 93.928; *p* < 0.001). Analysis of contrasts (Bonferroni-corrected) showed that the score of the 20 cm synchronous VHI (20S) resulted significantly greater than the asynchronous control condition (20A) (*z* = 9.692; *p* < 0.001) and that so did the 40 cm synchronous VHI (40S; *z* = 9.619; *p* < 0.001). The elongation effect was not found significant (Fig. 5, top-right graph; df = 1, 134.00; *F* = 0.004; *p* = 0.947). However, the order effect was found significant (Fig. 6, top graph; df = 2, 134.00; *F* = 7.247; *p* = 0.001). The analysis of contrasts showed that the VHI score of the first condition presented to the participant was significantly greater than both the second one (*z* = 3.386; *p* = 0.004) and the third one (*z* = 3.200; *p* = 0.008). No significant difference was found in the VHI score between the second and third condition (*z* = −0.184; *p* = 1.000).

10.1523/ENEURO.0244-23.2024.f5-1 Figure 5-1 Download Figure 5-1, TIF file.

The synchronicity effect on PD was found very close to being significant (Fig. 5, middle-left graph; df = 1, 160; *F* = 3.847; *p* = 0.052). The elongation effect was not found significant on PD (Fig. 5, middle-right graph; df = 1, 160; *F* = 0.837; *p* = 0.365). Nevertheless, PD resulted significantly greater than 0 following the synchronous conditions (20S, df = 54; *t* = 3.035; *p* = 0.004; 40S, df = 54; *t* = 3.499; *p* < 0.001) but not following the asynchronous one (20A, df = 54; *t* = 0.118; *p* = 0.906), and no significant difference of PD premeasures (pre-PM) was found neither between 20S and 20A (df = 54; *t* = −0.940; *p* = 0.704) nor between 40S and 20S (df = 54; *t* = 0.583; *p* = 1.000). A significant effect of order on PD (Fig. 6, middle graph; df = 2; 160; *F* = 3.944; *p* = 0.021) was highlighted. Contrasts revealed that the PD elicited by the first condition presented to participants was significantly greater than the PD elicited by the second condition (*z* = 2.770; *p* = 0.017) but not significantly greater than the PD elicited by the third condition (*z* = 1.787; *p* = 0.222). No significant difference was found in PD between the second and third condition (*z* = −0.982; *p* = 0.978).

No significant effect of synchronicity was found on ΔPSE (Fig. 5, bottom-left graph; df = 1; 190; *F* = 1.517; *p* = 0.220). There was a significant effect of elongation (Fig. 5, bottom-right graph; df = 1; 190; *F* = 5.223; *p* = 0.023). The contrasts however showed no significant difference in ΔPSE neither between 40S and 20A (*z* = −1.056; *p* = 1.000) nor between 40S and 20S (*z* = −2.285; *p* = 0.111). ΔPSE was found significantly <0 following the 40S condition but not for other conditions (20A, df = 64; *t* = −1.717; *p* = 0.091; 20S, df = 64; *t* = 0.316; *p* = 0.753; 40S, df = 64; *t* = −2.295; *p* = 0.025), and no significant difference of PSE premeasures (pre-PSE) was found neither between 20S and 20A (*z* = −1.101; *p* = 0.544) nor between 40S and 20S (*z* = 0.186; *p* = 1.000). Nevertheless, there was a significant effect of order on ΔPSE (Fig. 6, bottom graph; df = 2; 192; *F* = 12.015; *p* < 0.001). The contrasts showed that the ΔPSE was significantly lower after the first condition than both the second condition (*z* = −4.070; *p* < 0.001) and the third condition (*z* = −4.398; *p* < 0.001). No significant difference was found in ΔPSE between the second and third condition (*z* = −0.337; *p* = 1.000). A significant correlation was found between RHI index and PD (Fig. 8*A*, top graph; *ρ* = 0.187; *p* = 0.016), but neither between RHI and ΔPSE (Fig. 8*A*, bottom-left graph; *ρ* = −0.125; *p* = 0.094) nor between PD and ΔPSE (Fig. 8*A*, bottom-right graph; *r* = −0.056; *p* = 0.488).

### First-condition analysis

**Figure 7.**
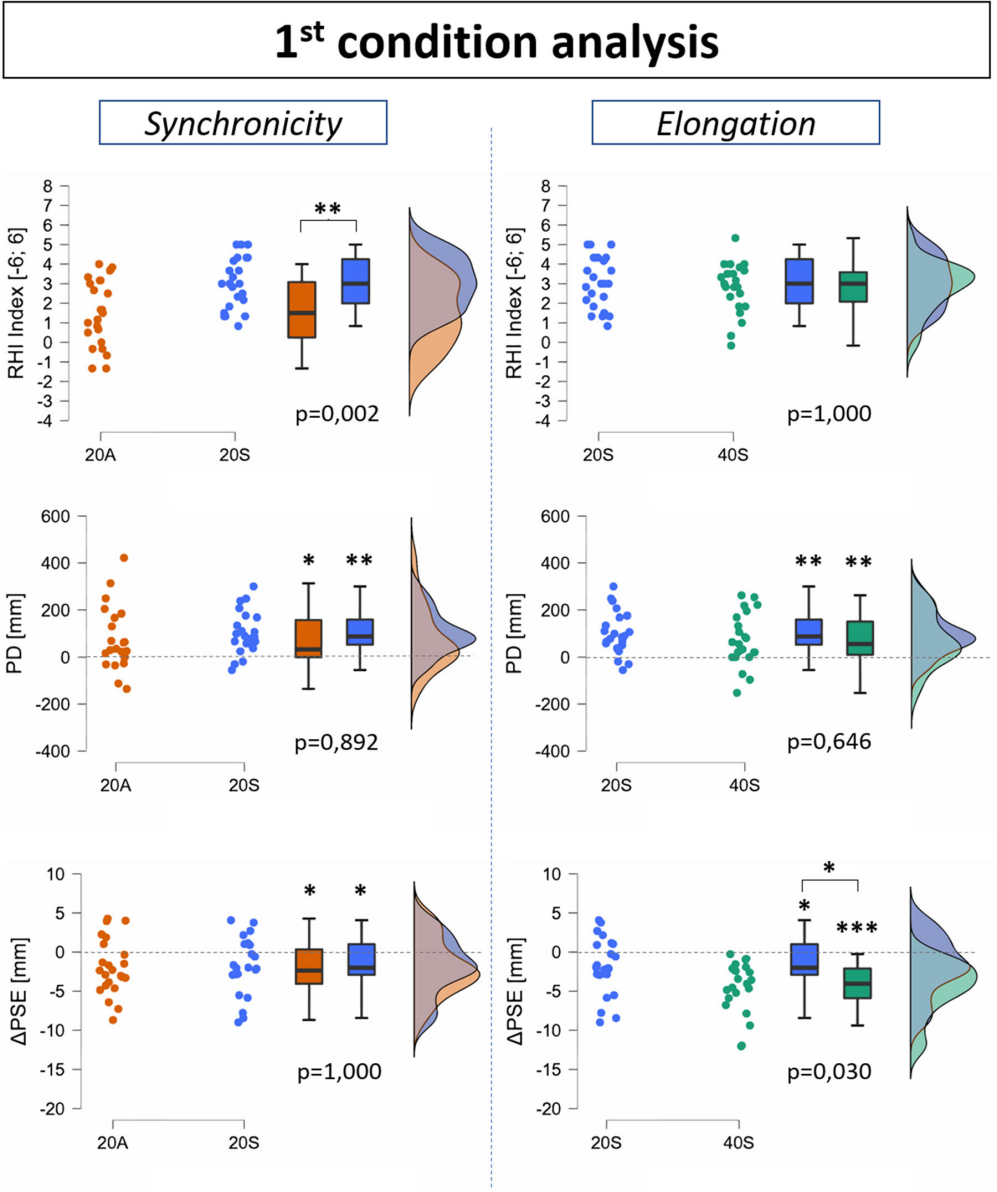
Raincloud plots showing results of the first-condition analysis on the synchronicity effect (left) and elongation effect (right) on the RHI index (top), PD (middle), and ΔPSE (bottom). Asterisks on top of a singular box plot (without brackets) indicate the significance level of the difference from 0. Asterisks on top of brackets indicate the significance level of the difference between the indicated conditions, and the corresponding *p* value is displayed below the box plots. Asterisks meanings: *p < 0.05; **p < 0.01; ***p < 0.001. Extended data related to the ΔEA is displayed in Extended Data Figure 7-1.

The synchronicity effect was found significant on the RHI index (Fig. 7, top-left graph). Indeed, the score of the synchronous VHI resulted significantly greater than the asynchronous control condition (20S vs 20A, *t* = 3.473; *p* = 0.002), and no significant effect of elongation was found on the RHI index (Fig. 7, top-right graph; 20S vs 40S, *t* = 0.606; *p* = 1.000).

10.1523/ENEURO.0244-23.2024.f7-1 Figure 7-1 Download Figure 7-1, TIF file.

No significant difference of PD premeasures was found between conditions (20S vs 20A, *t* = −0.004; *p* = 1.000; 40S vs 20S, *t* = 1.063; *p* = 0.588), and all conditions were found to elicit a PD significantly >0 (Fig. 7, middle line graphs; 20A, *t* = 2.737; *p* = 0.012; 20S, *t* = 3.547; *p* = 0.002; 40S, *t* = 3.116; *p* = 0.005). However, no significant effect of synchronicity nor elongation was found on PD (20S vs 20A, *t* = 0.770; *p* = 0.892; 40S vs 20S, *t* = −0.999; *p* = 0.646).

**Figure 8.**
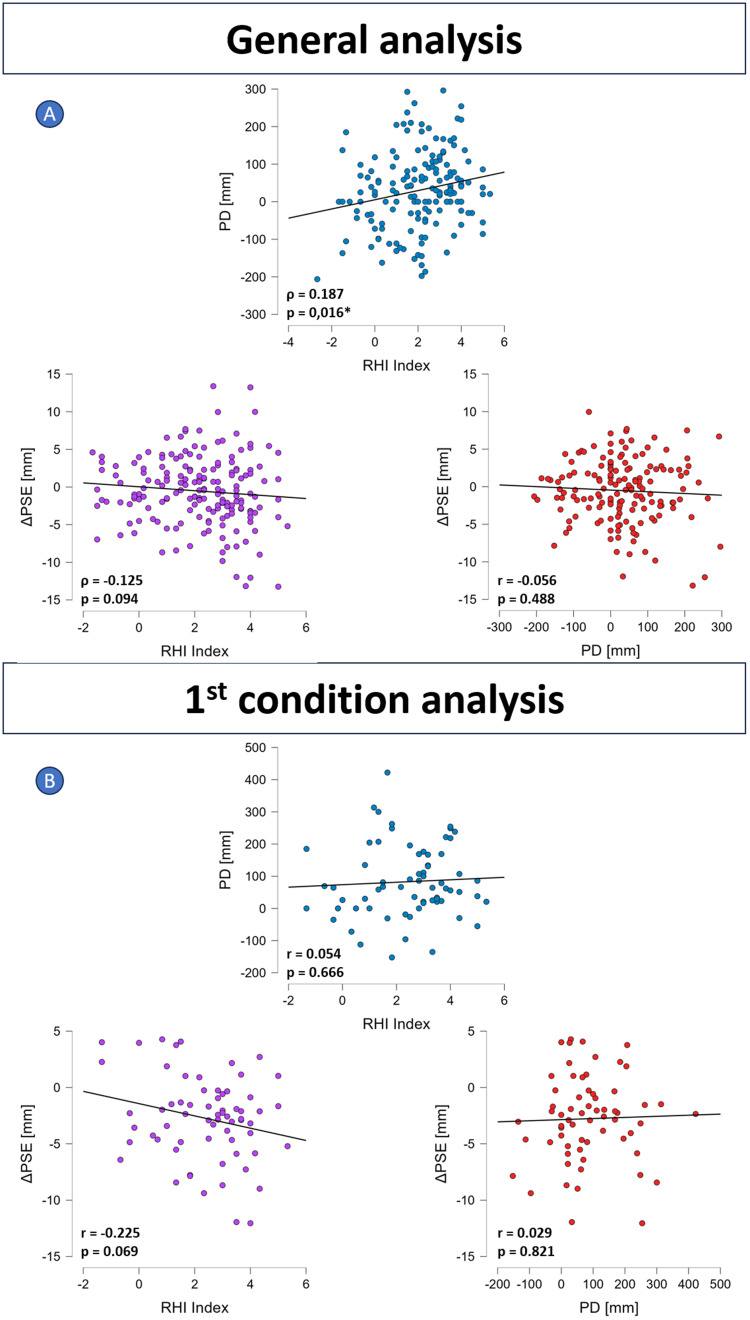
Correlation plots for the general analysis (***A***) and for the analysis on the first condition (***B***): RHI index and PD (top), RHI index and ΔPSE (bottom-left), PD and ΔPSE (bottom-right). *ρ* and *r* represent Spearman's *ρ* and Pearson's *r*, respectively. Asterisk meanings: *p < 0.05.

No significant difference of PSE premeasures (pre-PSE) was found between conditions 20S and 20A (*t* = 1.463; *p* = 0.302) nor between 40S and 20S (*t* = 1.029; *p* = 0.620). All conditions were found to elicit a ΔPSE significantly <0 (Fig. 7, bottom line graphs; 20A, *t* = −2.608; *p* = 0.016; 20S, *t* = −2.288; *p* = 0.033; 40S, *t* = −6.232; *p* < 0.001). No significant effect on synchronicity was found on ΔPSE (20S vs 20A, *t* = 0.096; *p* = 1.000), but the elongation effect was significantly present (40S vs 20S, *t* = −2.551; *p* = 0.030). The correlation between RHI index and ΔPSE was found close to significance (Fig. 8*B*, bottom-left graph; *r* = −0.225; *p* = 0.069), and no significant correlation was found neither between RHI index and PD (Fig. 8*B*, top graph; *r* = 0.054; *p* = 0.666) nor between PD and ΔPSE (Fig. 8*B*, bottom-right graph; *r* = 0.029; *p* = 0.821).

## Discussion

The aim of the study was to understand whether the change in TDP following a bodily illusion demonstrated by previous studies (Taylor-Clarke et al., 2004; De Vignemont et al., 2005) could take place through embodiment over a fake limb. We used a VHI paradigm with an elongated forearm in a 1PP virtual environment to induce the bodily illusion of owning an elongated forearm and assessed the resulting change in subjective perception of distance between two simultaneously touched points using a TDPT. Through a LMM, we investigated on each of our outcomes (RHI index, PD, ΔPSE) the effects of the synchronicity of the VHI, of the forearm elongation, and of the order in which the conditions (20A, 20S, and 40S) were presented to participants. We also investigated the correlation between different aspects of the elongated forearm embodiment and the changes in TDP. The embodiment illusion proved effective, as the effect of synchronicity was found significant with participants perceiving a significantly higher level of ownership (from the RHI index) after the synchronous VHI (20S and 40S) compared with the asynchronous condition (20A). Our experiment confirms previous findings on the effectiveness of synchronous visuotactile stimulation in eliciting ownership over a virtual limb (Slater et al., 2008; Pyasik et al., 2020). Besides, no effect of the forearm elongation on the perceived ownership was highlighted as the elongation effect did not result significant, meaning that both elongations resulted in a similar embodiment level. This comes in line with previous results showing that it is possible to embody a fake hand placed farther than the real one along the distal plane with a RHI or VHI paradigm (Armel and Ramachandran, 2003; Kilteni et al., 2012; Kalckert et al., 2019). More specifically, previous study (Kilteni et al., 2012) showed a steady ownership level with a virtual forearm of one, two, and three times the normal forearm length. Considering that the elongation magnitudes (20 and 40 cm elongations) of our study are comprised in these lengths (forearm length of participants, 25.77 ± 2.22 cm; thus a times-three forearm length would be equivalent to a 50 cm elongation), our results confirm the finding of that study.

From the general analysis, synchronous conditions (20S and 40S) caused a significant drift of the perceived position of the hand from the one perceived prior to the VHI toward the position of the virtual hand visualized during the VHI, whereas the asynchronous condition (20A) did not. This result would hint toward an effect of the synchronicity of the VHI on the PD with a higher PD due to the synchronous VHI with respect to the asynchronous VHI. Nevertheless, although very close to significance, the VHI synchronicity was not found to influence the PD, neither was the forearm elongation magnitude. Mixed results were found considering the change in TDP. On the one hand, as no significant effect of synchronicity was found, the VHI synchronicity did not prove to influence the change in TDP. This comes in opposition with our first hypothesis on the causal link between the synchronous visuotactile stimulation and the increase in TDP (decrease of the PSE). On the other hand, as shown by the LMM analysis, the elongation effect proved significant on ΔTDP (significant main effect of the elongation). Furthermore, the greater elongation condition (40S) significantly increased the TDP (significantly decreased the PSE) with respect to the pre-VHI perception, whereas smaller elongation conditions (20A and 20S) did not. This suggests the presence of an effect of the elongation magnitude of the virtual forearm, with greater increases in TDP for greater elongations.

Different factors could have affected our results. Testing multiple repetitions of the RHI paradigm successively could have decreased participant sensitivity to the illusion. Additionally, participants underwent all conditions successively without removing the headset nor moving their left arm. Therefore, no proper “reset” of the experimental conditions took place between the different conditions, and thus the effects of one condition might have been transferred to the successive condition. The analysis of the effect of the order in which the conditions were presented to the participants proves that those hypotheses are sound. Indeed, for all three outcomes, the order effect was found significant, and more specifically, the contrasts showed that all outcomes where significantly greater after the first condition than after the second and third condition (except for the third condition of the PD). Considering the fact that the aforementioned issues could have affected the second and third conditions undergone by participants but not of the first one and taking into account the crucial effect of the order in which the conditions were presented to the participants, the between-subject analysis on the first condition tested appears greatly relevant.

Regarding the perceived embodiment of the virtual hand through the VHI, the analysis on the first condition gave identical results as the previous analysis. However, the analysis on the first condition revealed that, after all VHI conditions, participants perceived the position of their real hand significantly drifted toward the position of the virtual hand (PD), independently from the VHI synchronicity or the magnitude of elongation. Similarly to PD findings, ΔPSE analysis showed that participants perceived an increase in TDP on the real forearm after all elongated VHI conditions and that, as previously suggested, a greater virtual forearm elongation results in a greater augmentation in TDP (ΔPSE due to 40S significantly lower than ΔPSE due to 20S). This means that the magnitude of virtual forearm elongation matters in determining the changes of TDP. On the other hand, the effect of synchronicity of the VHI on the modification of the TDP is absent. As expected, the significant difference in ΔPSE among the conditions of arm elongation means that the paradigm is able to modulate the tactile perception and, hence, the body schema. However, differently from studies performed in the real environment (Van Der Hoort et al., 2011; Bruno and Bertamini, 2010), we found no correlation between RHI index and ΔPSE induced by VR.

The presence of the elongation effect and the absence of a significant synchronicity effect, combined also with the absence of correlation between RHI index and ΔPSE, suggest that in our study, the modification of the TDP is not directly explained with the visuotactile integration elicited by the VHI paradigm. Although it is widely acknowledged that embodiment occurs through multisensory integration (Castro et al., 2023), the visual component (e.g., “visual capture phenomenon”; Pavani et al., 2000) alone may be sufficient to induce it; this effect in particular is reported in immersive 1PP virtual experience (Maselli and Slater, 2013; Tieri et al., 2015; Argelaguet et al., 2016; Bailey et al., 2016; Pavone et al., 2016; Spinelli et al., 2018; Bourdin et al., 2019; Matamala-Gomez et al., 2019). Here, the familiarization phase may have played a pivotal role; it is plausible that the sense of agency alone carried-over embodiment toward the virtual forearm (D’Angelo et al., 2018). In this perspective, given the strong impact of visual feedback on the subject's agency, visuotactile integration may not have played a primary role in incorporating the avatar's arm into the subjects’ body representation yielding no effect of synchronicity. In line with this, a previous study found changes in the perceived size of objects after the exposure to VR environment: participants experienced, in 1PP VR, vision and agency over a virtual hand of modified dimensions but without any form of visuotactile stimulation (Likenauger et al., 2013).

Although a significant effect of synchronicity was highlighted by the RHI index value, suggesting differences in embodiment levels between asynchronous and synchronous conditions, this does not necessarily indicate a disownership of the virtual hand in the asynchronous condition. In fact, many authors reported only reduced—but not null—illusion during the asynchronous condition (Ehrsson et al., 2004; Costantini and Haggard, 2007; Shimada et al., 2009). In addition, considering that the RHI questionnaire was an adapted version of the original one which was based on visuotactile integration (Botvinick and Cohen, 1998), the extracted index may not be capable to highlight the effect of visual capture on the avatar's embodiment.

As regards PD results, we found significant difference with respect to 0 of drift values for all conditions. Consistently with the aforementioned findings, the drift in perceived hand location may be mainly caused by the visual feedback from the elongated upper limb (Perez-Marcos et al., 2012). The absence of a virtual forearm elongation effect on the PD (no significant difference between 40S and 20S) is an intriguing finding. We could hypothesize that proprioception is malleable but bounded to some extent by our natural body schema (prior to its alteration) and insensitive to excessive distortions. Indeed, previous study found the PD to be significantly >0 with a virtual forearm of two and three times the length of the real limb length (equivalent to our 20 and 40 cm elongation, respectively) but without any significant increase of the elicited PD for the times-three condition with respect to the times-two condition (Kilteni et al., 2012). They furthermore found the PD effect to be seemingly limited to a times-three forearm length by finding an absence of PD for a times-four forearm length.

Both TDP and PD seem to be affected by body schema modifications (De Vignemont, 2010, 2011; Romano et al., 2015; Meraz et al., 2018). However, as shown by the significant effect of elongation, TDP seems to better highlight these changes. This may be due to the task resolution which makes TDP more suitable to measure perceptual changes, or, alternatively, it may disclose that exteroception and proprioception differently respond to our modulations.

In conclusion, in our everyday life, we interact with the environment using our body to sense. Like any sensor, our perception requires specific reference frames to measure parameters accurately. For instance, to perceive tactile distances, we rescale our perception based on the currently perceived dimensions of the touched body part. It is believed that rescaling process involves the implicit model of the metric properties of our body constructed by the brain: the body schema. Several studies have proven the latter by modifying the body schema of participants through own body size modification illusions and observing a correlated modification of the TDP. However, to our knowledge, none had investigated it following a modification of the body schema through the embodiment of an artificial (i.e., virtual) body part. In this study, we investigated the effects on TDP of a virtual elongated forearm experienced in 1PP virtual environment. Even though we found an increase of the TDP positively associated with the virtual forearm elongation magnitude, no link has been found between the perceived embodiment over the virtually elongated arm resulting from the visuotactile synchronicity of the VHI and the perceived augmentation of the TDP, suggesting that the alteration of the body schema has taken place mainly through the visual feedback over the elongated body part. We further hypothesized that a virtual embodiment due to the immersive 1PP experience of a virtual body might have taken place, dominating the VHI-induced embodiment.

## Synthesis

Reviewing Editor: Niko Busch, Westfalische Wilhelms-Universitat Munster Decisions are customarily a result of the Reviewing Editor and the peer reviewers coming together and discussing their recommendations until a consensus is reached. When revisions are invited, a fact-based synthesis statement explaining their decision and outlining what is needed to prepare a revision will be listed below. The following reviewer(s) agreed to reveal their identity: Mariano D'angelo, Marie Chancel.

# Synthesis

This manuscript was reviewed by two experts. Both reviews highlight the merits of your study, but they also agree on the need to improve the manuscript further. I encourage you to carefully address each of the reviewers' points. Specifically, they both request modifications of some of the conclusions or a deeper and more careful discussion of the design and conclusions.

Moreover, both reviewers comment on the statistical analysis, and one concern pertains to the analysis and interpretation of the null findings. I agree that it would be useful to include a Bayesian analysis to substantiate the evidence for the Null, and to include a measure of effect sizes or confidence intervals for all relevant analyses. While you do provide confidence intervals for the t-tests in Table 1, this would also be relevant for the correlations. A mixed linear regression model may be a useful alternative that does not require a large number of pairwise tests.

I would like to add the following points to the reviewers' comments:

- All t-values should be reported with t-statistics and degrees of freedom.

- What does the column titled "Power (95% C.I. for Cohen's d)" indicate? Power is a probability that should be bound between 0 and 1, but some of the values in this column take negative values or values larger than 1.

- Finally, I would like to ask you to consider making the data and code publicly available.

# Reviewer 1

In the present study, authors investigate if altering the body schema through a virtual hand illusion impacts the perception of tactile distances on the arm. To this end, participants performed a tactile distance task before and after authors induced an illusory ownership over an elongated virtual arm (20 cm or 40 cm elongations). Embodiment of the virtual arm, as measured with a questionnaire, was found significantly greater after the synchronous visuo-tactile stimulation condition compared to the asynchronous one. However, authors found that the elongated arm did not affect the tactile distance task significantly.

The topic is interesting, I have some major concerns:

Introduction:

1) I have some reservations about the statement that "when the environment is experienced through our body (i.e., in somatosensation), it has been suggested that the reference frame we employ is our body and its metric proprieties". I found similar statements also in the abstract. (e.g., "This raises the neuroscientific question of which reference frame is used by humans when exploring the environment. Previous studies suggested that, when using touch, the body employed as measuring tool also serves as reference frame"). These statements are surely true. However, in the current paper authors do not study tactile perception involved in estimating the size of external environmental stimuli. Rather, the tactile distance task is used as a proxy to gauge changes in the arm length representation (i.e., the body schema). I recommend that the authors consider rephrasing this sentence for clarity.

Methods

2) My biggest concern about the methodology is the avatar familiarization period, during which participants experience agency and ownership prior to the actual ownership manipulation through visuo-tactile stimulation. This could potentially lead to carryover effects that might contribute to the nuanced and inconclusive findings of the study (i.e., the absence of a significant differences in proprioceptive drift and the tactile distance effect between synchronous and asynchronous conditions). It worthy to notice that the sense of agency alone, without ownership, seems to be sufficient to create a modulation of the body schema (see for instance D'Angelo et al., 2018).

3) In the current study, the asynchronous visuo-tactile condition seems to me more a "disownership condition" where participants disowned the avatar hand that has been previously owned during the familiarization period.

4) The specific arm length used during the familiarization phase remains unclear. Is it tailored to each participant's actual arm length, or is it based on an average length?

5) Is there a particular reason for the exclusion of the 40 cm elongated arm in the asynchronous condition? It's reasonable to think that a 20 cm elongation might not induce significant differences in the updating of the body schema. This could be why there were no significant differences observed between the 20 cm synchronous and asynchronous conditions, or even between the two synchronous conditions (20 cm vs 40 cm). One potential approach could involve comparing the Delta PSE between the 20 cm Asynchronous and 40 cm Synchronous conditions.

6) Linked to the previous point, were participants able to detect the 20 cm elongation following the familiarization phase?

Results

7) I propose that authors clarify their expectations regarding the TDPT when arm representation length increases. From my perspective, I anticipate that a certain distance is perceived shorter when the arm representation is longer (similar to Canzoneri et al., 2013). Is this understanding accurate?

8) I think that results section would be much clearer if authors elucidate which direction of PSE change aligns with an elongation of the arm representation. Given that the sigmoid function is fitted based on the proportion of "forearm larger" responses, a higher PSE value implies that participants require a greater distance to perceive the distances on the forearm as larger. Consequently, I presume that a lower PSE value signifies an elongated arm representation (i.e., the distance is perceived shorter after an arm elongation). Is my interpretation correct?

9) I generally found the comparison against 0 a bit unnatural. I am not saying is wrong and I know it is an established procedure, but it strikes me as less ecologically valid (considering the feasibility of a true zero change). Prior to conducting this comparison, I would like to propose authors to implement a Bayesian analysis between synchronous and asynchronous conditions.

# Reviewer 2

The authors introduce nicely their question about how the embodiment of a visually altered limb may lead to a change in body schema. They further hypothesize that this change can be observed via a tactile distance perception (TDP) change. While the introduction is clear, the method well explained and the question interesting, I have major concerns, especially regarding data analysis and the interpretation of the results.

As far as I understood, the initial idea behind the study can be simplified as follows: "A change in body schema leads to a change in TDP; Let's alter the body schema using virtual hand illusion (VHI)". Then the authors do not find a link between the VHI index and TDP, and still they conclude that the observed change in TDP with one of their factors is caused by a change in body schema. Thus, it reads like they are validating their hypothesis even though their experimental predictions did not occur, by relying on a post-hoc notion of virtual embodiment. I don't see how the present results can afford the final sentence of the manuscript "TDP revealed a modification of the body schema that the PD did not detect, suggesting that tactile exteroception and proprioception are affected differently by body schema modifications." Then, the virtual embodiment is presented in opposition to visuotactile embodiment, which I find surprising. Shouldn't the 2 add up? How would the authors collect virtual embodiment independently from the one induced by visuotactile synchrony?

My concern is about the methodological choice made by the authors:

- First, I regret that the authors did not use a factorial design (20A 20S 40A 40S). Nonetheless, a mixed-model regression is still possible and, in my opinion, preferable to the current multiplication of t.test, even if these are corrected. Such an approach could also include an ORDER factor, to account for the order in which the participants underwent the conditions without losing 2 third of the acquired data. It would also allow better illustration of the analysis. As it is, because of the random jitter, the blue dots in figure 5 looks like they correspond to different data.

- Effect sizes are necessary to interpret the results correctly, especially given the number of nonsignificant results not in line with the authors' predictions.

- Second, if I understood correctly, the psychometric curves have been extracted from only 4 repetitions of each stim (2 times per body site, once 1st, once 2nd) and without any optimization techniques. I question the robustness of the correcting PSE assessment. Have the authors thought about using a more robust fitting procedure? For example using a toolbox such as Palamedes (Prins &Kingdom, 2018).

My second concern is about the methodological choice made by the authors:

- First, I regret that the authors did not use a factorial design (20A 20S 40A 40S). Nonetheless, a mixed-model regression is still possible and, in my opinion, preferable to the current multiplication of t.test, even if these are corrected. Such an approach could also include an ORDER factor, to account for the order in which the participants underwent the conditions without losing 2 third of the acquired data. It would also allow better illustration of the analysis. As it is, because of the random jitter, the blue dots in figure 5 looks like they correspond to different data.

- Effect sizes are necessary to interpret the results correctly, especially given the number of nonsignificant results not in line with the authors' predictions.

- Second, if I understood correctly, the psychometric curves have been extracted from only 4 repetitions of each stim (2 times per body site, once 1st, once 2nd) and without any optimization techniques. I question the robustness of the correcting PSE assessment. Have the authors thought about using a more robust fitting procedure? For example using a toolbox such as Palamedes (Prins &Kingdom, 2018).

Minor:

- The references van der Hoort et al. 2011 and Bruno &Bertamini 2010 (p.22) should, in my opinion, be mentioned as early as in the introduction since their experimental questions and results are so closely related to the present study. The authors may also want to discuss Maselli et al (2016, The sense of body ownership relaxes temporal constraints for multisensory integration).

- P10: I think the authors could be clearer in explaining the meaning of the PSE and of its changes by explaining first what a positive PSE means (TDP greater in one body site) and then what reduction of the PSE means.

- Page 12 and 13: both sections 2.4.1 and 2.4.2. start with "all data (RHI Index, PD and ΔPSE) were distributed normally" and end with an example of non-normal data for correlation analyses. Unless I missed something, there's a contradiction here.

- I would avoid the abbreviation FA for forearm when using psychophysics, it is usually used for false alarms.

- Out of curiosity, have you looked at the impact of your conditions on EA? Have you also checked the correlation RHI index/PD?

- P 16: I think there's a typo: the pre-measures concern PSE and not delta(PSE)

## Author Response

Manuscript Number: eN-NWR-0244-23 Experiencing an elongated limb in virtual reality modifies the tactile distance perception of the corresponding real limb The revised version of the manuscript entitled "Experiencing an elongated limb in virtual reality modifies the tactile distance perception of the corresponding real limb" is updated. We wish to thank the Reviewers for the time spent on our manuscript. We carefully considered the Reviewers' observations and suggestions that helped us to refine the quality of the draft. Point-by-point replies to the comments of editor (QE) and reviewers (QR) are provided below. For clarity in our answers, some of the questions have been divided in two and answered separately and changes to the text are underlined here. Sincerely, on behalf of all co-authors. # Editor This manuscript was reviewed by two experts. Both reviews highlight the merits of your study, but they also agree on the need to improve the manuscript further. I encourage you to carefully address each of the reviewers' points. Specifically, they both request modifications of some of the conclusions or a deeper and more careful discussion of the design and conclusions. QE1: Moreover, both reviewers comment on the statistical analysis, and one concern pertains to the analysis and interpretation of the null findings. I agree that it would be useful to include a Bayesian analysis to substantiate the evidence for the Null. AE1. We conducted a Bayesian analysis on all our comparisons, but results were similar to the former analysis and we selected to not include in the manuscript. Please see also answer AR1.10 for details. QE2: I agree to include a measure of effect sizes or confidence intervals for all relevant analyses. While you do provide confidence intervals for the t-tests in Table 1, this would also be relevant for the correlations. AE2. Effect sizes of all measures were added to the results section. QE3: A mixed linear regression model may be a useful alternative that does not require a large number of pairwise tests. AE3. Following the suggestion of the reviewer 2, we used a mixed-model regression model. We also included the ORDER factor to account for the effect of the order in which participants underwent the conditions. The obtained results were in line with our previous analyses. Please see also answer AR2.2 for details. I would like to add the following points to the reviewers' comments: QE4: All t-values should be reported with t-statistics and degrees of freedom. AE4. All t-statistics and degrees of freedom were added. QE5: What does the column titled "Power (95% C.I. for Cohen's d)" indicate? Power is a probability that should be bound between 0 and 1, but some of the values in this column take negative values or values larger than 1. AE5. The title of this column was a typo. The statistics tables were reworked based on the new LMM analyses. QE6: Finally, I would like to ask you to consider making the data and code publicly available. AE6. We agree to make the data and code publicly available. We are currently working on the repository on which to post the data and commenting our code to make it easier to understand. # Reviewer 1 In the present study, authors investigate if altering the body schema through a virtual hand illusion impacts the perception of tactile distances on the arm. To this end, participants performed a tactile distance task before and after authors induced an illusory ownership over an elongated virtual arm (20 cm or 40 cm elongations). Embodiment of the virtual arm, as measured with a questionnaire, was found significantly greater after the synchronous visuo-tactile stimulation condition compared to the asynchronous one. However, authors found that the elongated arm did not affect the tactile distance task significantly. The topic is interesting, I have some major concerns: Introduction: Q1.1: I have some reservations about the statement that "when the environment is experienced through our body (i.e., in somatosensation), it has been suggested that the reference frame we employ is our body and its metric proprieties". I found similar statements also in the abstract. (e.g., "This raises the neuroscientific question of which reference frame is used by humans when exploring the environment. Previous studies suggested that, when using touch, the body employed as measuring tool also serves as reference frame"). These statements are surely true. However, in the current paper authors do not study tactile perception involved in estimating the size of external environmental stimuli. Rather, the tactile distance task is used as a proxy to gauge changes in the arm length representation (i.e., the body schema). I recommend that the authors consider rephrasing this sentence for clarity. A1.1. We thank the reviewer for this comment, and we apologize that these statements appeared to be misleading. However, although here the tactile distance task is used as proxy to measure changes in arm representation, subjects are asked to estimate the distance between two real points resulting from different forks measures, which are considered external environmental stimuli. In our study, external stimuli (forks) are used to infer changes in body representation, but they are still part of the subject's perceptual evaluation. Of course, we edit the abstract and introduction: Abstract: "In this way, we use the TDP task as a proxy to gauge changes in the body schema" Introduction: "This hypothesis is sound because it has been widely shown that the brain integrates a higher-order reconstruction of the body that is an implicit model of its metric properties (Longo and Haggard, 2010, 2012), namely the body schema (De Vignemont, 2010), and of the space where the interaction occurs. This means also that change in body schema could affect the perception of the environment." Methods Q1.2: My biggest concern about the methodology is the avatar familiarization period, during which participants experience agency and ownership prior to the actual ownership manipulation through visuo-tactile stimulation. This could potentially lead to carryover effects that might contribute to the nuanced and inconclusive findings of the study (i.e., the absence of a significant differences in proprioceptive drift and the tactile distance effect between synchronous and asynchronous conditions). It worthy to notice that the sense of agency alone, without ownership, seems to be sufficient to create a modulation of the body schema (see for instance D'Angelo et al., 2018). A1.2. We thank the reviewer for this relevant comment and insightful reference. We indeed used the familiarization phase to provide participants with the sense of agency over the avatar. Anyway, we believed that since no elongation of the avatar's forearm was applied at that point, it would not modulate the participants' body schema. However, such phase could have a pivotal role in embodying the avatar and minimizing the effect of the synchronicity on our study. This aspect is now treated in Discussion. "Here, the familiarization phase may have played a pivotal role, it is plausible that the sense of agency alone carried-over embodiment towards the virtual forearm (D'Angelo et al., 2018). In this perspective, given the strong impact of visual feedback on the subject's agency, visuo-tactile integration may not have played a primary role in incorporating the avatar's arm into the subjects' body representation yielding no effect of synchronicity." Q1.3 In the current study, the asynchronous visuo-tactile condition seems to me more a "disownership condition" where participants disowned the avatar hand that has been previously owned during the familiarization period. A1.3. Thank you for your comment. It is of interest to note that previous studies highlighted that reported only reduced - but not null - illusion during the asynchronous condition (Ehrsson et al., 2004; Tsakiris and Haggard, 2005; Costantini and Haggard, 2007; Shimada et al., 2009), For such reason, we do not consider this condition as a disownership condition, rather a condition with lower level of embodiment. In addition, we hypothesize that visuo-tactile integration may have a minor role with respect to the vision of body avatar in 1PP virtual experience in incorporating the avatar's arm into the subjects' body representation reducing the effect of synchronicity on embodiment of the avatar. This is better discussed in Discussion section. "Despite a significant effect of synchronicity was highlighted by the RHI index value, suggesting differences in embodiment levels between asynchronous and synchronous conditions, this does not necessarily indicate a disownership of the virtual hand in the asynchronous condition. In fact, many authors reported only reduced - but not null - illusion during the asynchronous condition" Ehrsson, H. H., Spence, C., and Passingham, R. E. (2004). That's my hand! activity in premotor cortex reflects feeling of ownership of a limb. Science 305, 875-877. doi: 10.1126/science.1097011 Shimada, S., Fukuda, K., and Hiraki, K. (2009). Rubber hand illusion under delayed visual feedback. PLoS One 4:e6185. doi: 10.1371/journal.pone.0006185 Costantini, M., and Haggard, P. (2007). The rubber hand illusion: sensitivity and reference frame for body ownership. Conscious. Cogn. 16, 229-240. doi: 10.1016/j.concog.2007.01.001 Q1.4: The specific arm length used during the familiarization phase remains unclear. Is it tailored to each participant's actual arm length, or is it based on an average length? A1.4. The arm length used during the familiarization phase is based on an average arm length on literature (Greiner 1991). Greiner, T.M., 1991. Hand anthropometry of US army personnel (pp. 1-434). Natick, MA: US Army Natick Research, Development &Engineering Center. Q1.5: Is there a particular reason for the exclusion of the 40 cm elongated arm in the asynchronous condition? A1.5. We selected to exclude 40A condition because in our experimental protocol we had already a condition capable to elicit lower level of ownership thus no expected modulation of the body schema (20A), and we wanted to limit the repetition and duration of the experiment. Indeed, the embodiment induced by the RHI procedure suffers of habituation (Convento, Romano, Maravita, &Bolognini, 2018; Bekrater-Bodmann, Foell, Diers, &Flor, 2012) and too many repetitions of the VHI would minimize its effect over time. Furthermore, we wanted to limit the experiment duration to one hour and a half considering participants' comfort and, hence, the reliability of the collected data. All the considerations taken together made us decide not to include a 40A condition. These motivations are reported also on the Method section. "We included only a single common control condition based on asynchronous tactile stimulation of the virtual hand. This experimental choice did not allow to apply a balanced factorial design to our study. However, this choice has been done to reduce the duration of the experimental sessions for participant comfort and, consequently, reliability of the collected data and preventing the habituation to VHI." Convento, S., Romano, D., Maravita, A. and Bolognini, N., 2018. Roles of the right temporo‐ parietal and premotor cortices in self‐location and body ownership. European Journal of Neuroscience, 47(11), pp.1289-1302. Bach, F., Cakmak, H., Maass, H., Bekrater-Bodmann, R., Foell, J., Diers, M., Trojan, J., Fuchs, X. and Flor, H., 2012. Illusory hand ownership induced by an MRI compatible immersive virtual reality device. Biomedical Engineering/Biomedizinische Technik, 57(SI-1-Track-L), pp.718-720. Q1.6: It's reasonable to think that a 20 cm elongation might not induce significant differences in the updating of the body schema. This could be why there were no significant differences observed between the 20 cm synchronous and asynchronous conditions, or even between the two synchronous conditions (20 cm vs 40 cm). One potential approach could involve comparing the Delta PSE between the 20 cm Asynchronous and 40 cm Synchronous conditions. A1.6. As regards the performed analysis, although this experimental choice did not allow to apply a balanced factorial design, a mixed-model regression is still possible and following the suggestion of the reviewer 2 (Q2.3.), we used a mixed-model regression model including the ORDER factor to account for the effect of the order in which participants underwent the conditions. The obtained results were in line with our previous analyses regarding the synchronicity effect but showed a significant effect of elongation of the ΔPSE. Q1.7: Linked to the previous point, were participants able to detect the 20 cm elongation following the familiarization phase? A1.7. We did not systematically ask participants if they were able to perceived the specific 20cm elongation following the familiarization phase. However, when discussing with participants about the experiment they underwent, they reported that were aware of the two different elongations and that the position of the virtual hand was different with respect to their actual position. Results Q1.8: I propose that authors clarify their expectations regarding the TDPT when arm representation length increases. From my perspective, I anticipate that a certain distance is perceived shorter when the arm representation is longer (similar to Canzoneri et al., 2013). Is this understanding accurate? AR1.8. We indeed found the results of the study presented by (Canzoneri et al., 2013) very interesting. However, when establishing our hypotheses, we rather considered the results showed by (Taylor-Clarke et al., 2004) and (De Vignemont et al., 2005). They both show an increase in TDP (corresponding to a decrease in ΔPSE) after a visual and proprioceptive (respectively) bodily illusion of increased body parts dimensions. Those two studies are similar to ours in the sense that in all three cases a bodily illusion is used, whereas Canzoneri and colleagues study the effect of tool-use. Tool-use and bodily illusion might affect the body schema differently. We clarified our expectations in the introduction. The reference to (Canzoneri et al., 2013) was added. "Following the findings of (Taylor-Clarke et al., 2004) and (De Vignemont et al, 2005) who used bodily illusion and found that increased body size perception increases TDP, in contrast with (Canzoneri et al, 2013) who showed that tool-use reduces TDP, we hypothesized that the synchronous brush-stroking of a hand at the end of an elongated forearm would have increased the TDP on the corresponding real forearm and that the variation in TDP would have positively correlated with the amount of forearm elongation and with the achieved level of embodiment of the hand." Q1.9: I think that results section would be much clearer if authors elucidate which direction of PSE change aligns with an elongation of the arm representation. Given that the sigmoid function is fitted based on the proportion of "forearm larger" responses, a higher PSE value implies that participants require a greater distance to perceive the distances on the forearm as larger. Consequently, I presume that a lower PSE value signifies an elongated arm representation (i.e., the distance is perceived shorter after an arm elongation). Is my interpretation correct? AR1.9. As this was mentioned by both reviewers, we reworked and added explanations on the meaning of the PSE and of its sign. The sentence is here reported: "The TDP was measured by the Point of Subjective Equality (PSE, in millimeters). As clearly defined in (Vidotto et al., 2019), "the point of subjective equality is any of the points along a stimulus dimension (here ΔL) at which a variable stimulus (here forearm stimulation distance) is judged by an observer to be equal to a standard stimulus (here forehead stimulation distance)". If the PSE is positive, it means that the stimulation distance on the forearm has to be greater (by "PSE" millimeters) than the stimulation distance on the forehead to be felt as equal to the stimulation distance on the forehead, and vice versa for negative PSE. Thus, a reduction of PSE means the actual forearm stimulation distance has to be "less larger" than the forehead stimulation than before to be felt equal to the forehead stimulation, hence meaning that is was perceived larger." Q1.10: I generally found the comparison against 0 a bit unnatural. I am not saying is wrong and I know it is an established procedure, but it strikes me as less ecologically valid (considering the feasibility of a true zero change). Prior to conducting this comparison, I would like to propose authors to implement a Bayesian analysis between synchronous and asynchronous conditions. AR1.10. We conducted a Bayesian analysis on all our comparisons. The results were similar the former analysis and thus no different conclusion could be drawn. We present in the following tables the results of the Bayesian analyses. Statistical Table 1 Outcome Comparison BF10 Error % p-value RHI Index 20S vs 20A
