# Supplementary material for: Experiencing an Elongated Limb in Virtual Reality Modifies the Tactile Distance Perception of the Corresponding Real Limb
Source: eNeuro. 2024 Jun 14;11(6):ENEURO.0244-23.2024. doi: 10.1523/ENEURO.0244-23.2024 (PMC11208980; doi:10.1523/ENEURO.0244-23.2024)
Supplement: Table 2-1 — Download Table 2-1, DOCX file. [file eneuro-11-ENEURO.0244-23.2024-s005.docx]

| **Outcome** | **Effect** | **df** | **F** | **p-values** |
| --- | --- | --- | --- | --- |
| ΔEA | Synchronicity | 1, 202 | 3.677 | 0.057 |
| ΔEA | Elongation | 1, 202 | 0.447 | 0.504 |
| ΔEA | Order | 2, 202 | 1.091 | 0.338 |

***Table 2-1***
