# Supplementary material for: Experiencing an Elongated Limb in Virtual Reality Modifies the Tactile Distance Perception of the Corresponding Real Limb
Source: eNeuro. 2024 Jun 14;11(6):ENEURO.0244-23.2024. doi: 10.1523/ENEURO.0244-23.2024 (PMC11208980; doi:10.1523/ENEURO.0244-23.2024)
Supplement: Table5-1 — Download Table5-1, DOCX file. [file eneuro-11-ENEURO.0244-23.2024-s002.docx]

| **Outcome** | **Comparison** | **t** | **df** | **Cohen’s d** | **95% CI**  **(Lower)** | **95% CI**  **(Upper)** | **p-value** |
| --- | --- | --- | --- | --- | --- | --- | --- |
| ΔEA | 20S vs 20A | 1.592 | 44 | 0.469 | -0.119 | 1.053 | 0.119 |
| ΔEA | 40S vs 20A | -0.824 | 44 | 0.243 | -0.822 | 0.338 | 0.414 |

***Table 5-1***
