# Supplementary figures and images for: Experiencing an Elongated Limb in Virtual Reality Modifies the Tactile Distance Perception of the Corresponding Real Limb
Source: eNeuro. 2024 Jun 14;11(6):ENEURO.0244-23.2024. doi: 10.1523/ENEURO.0244-23.2024 (PMC11208980; doi:10.1523/ENEURO.0244-23.2024)

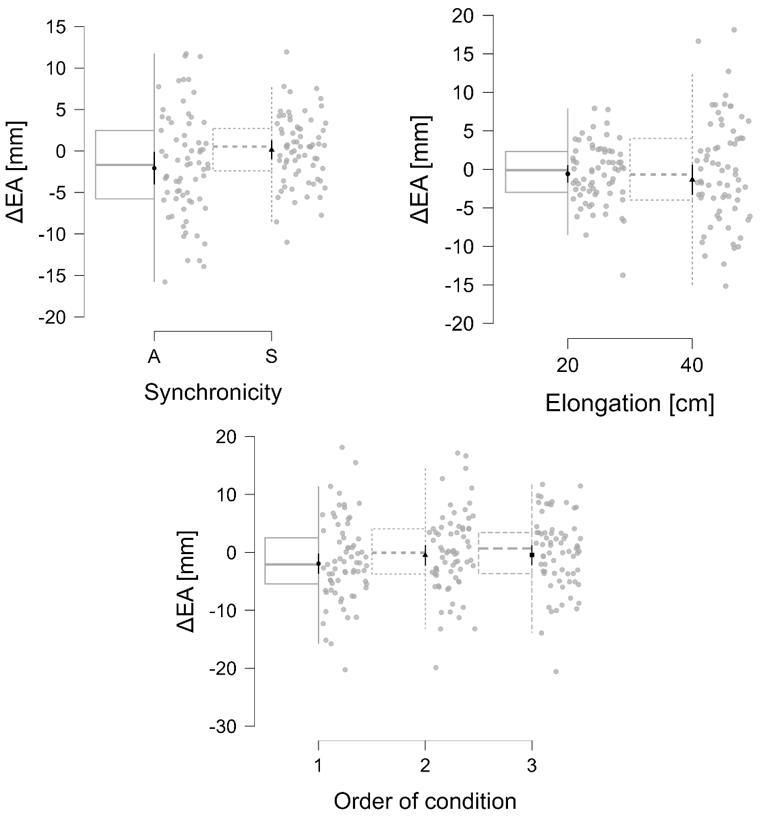

Supplement: Figure 5-1 — Download Figure 5-1, TIF file. [file eneuro-11-ENEURO.0244-23.2024-s003.tif]

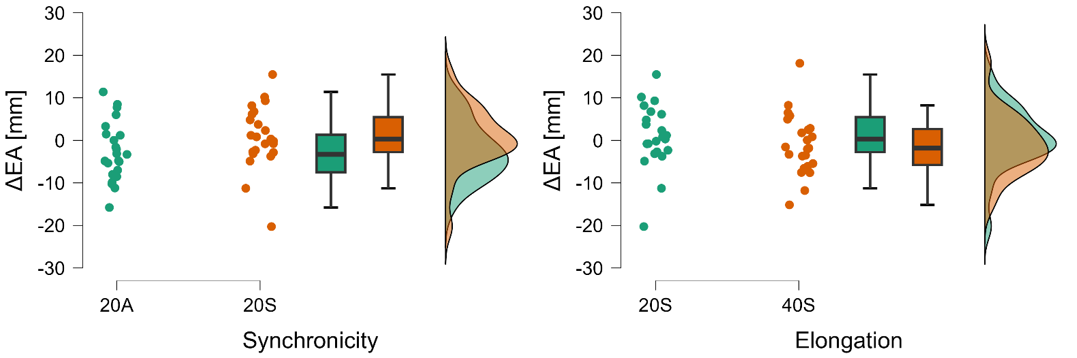

Supplement: Figure 7-1 — Download Figure 7-1, TIF file. [file eneuro-11-ENEURO.0244-23.2024-s004.tif]
